# Strategic Development of *Aurantiochytrium* sp. Mutants With Superior Oxidative Stress Tolerance and Glucose-6-Phosphate Dehydrogenase Activity for Enhanced DHA Production Through Plasma Mutagenesis Coupled With Chemical Screening

**DOI:** 10.3389/fnut.2022.876649

**Published:** 2022-04-26

**Authors:** Yusuf Nazir, Pranesha Phabakaran, Hafiy Halim, Hassan Mohamed, Tahira Naz, Aidil Abdul Hamid, Yuanda Song

**Affiliations:** ^1^Colin Ratledge Center for Microbial Lipids, School of Agricultural Engineering and Food Science, Shandong University of Technology, Zibo, China; ^2^Department of Food Sciences, Faculty of Science and Technology, Universiti Kebangsaan Malaysia, Bangi, Malaysia; ^3^Innovation Centre for Confectionery Technology (MANIS), Faculty of Science and Technology, Universiti Kebangsaan Malaysia, Bangi, Malaysia; ^4^Department of Botany and Microbiology, Faculty of Science, Al-Azhar University, Assiut, Egypt; ^5^Department of Bioscience and Biotechnology, Universiti Kebangsaan Malaysia, Bangi, Malaysia

**Keywords:** thraustochytrids, plasma mutagenesis, chemical screening, DHA production, zeocin, polydatin, *Aurantiochytrium*

## Abstract

Thraustochytrids, such as *Aurantiochytrium* and *Schizochytrium*, have been shown as a promising sustainable alternative to fish oil due to its ability to accumulate a high level of docosahexaenoic acid (DHA) from its total fatty acids. However, the low DHA volumetric yield by most of the wild type (WT) strain of thraustochytrids which probably be caused by the low oxidative stress tolerance as well as a limited supply of key precursors for DHA biosynthesis has restricted its application for industrial application. Thus, to enhance the DHA production, we aimed to generate *Aurantiochytrium* SW1 mutant with high tolerance toward oxidative stress and high glucose-6 phosphate dehydrogenase (G6PDH) activities through strategic plasma mutagenesis coupled with chemical screening. The WT strain (*Aurantiochytrium* sp. SW1) was initially exposed to plasma radiation and was further challenged with zeocin and polydatin, generating a mutant (YHPM1) with a 30, 65, and 80% higher overall biomass, lipid, and DHA production in comparison with the parental strains, respectively. Further analysis showed that the superior growth, lipid, and DHA biosynthesis of the YHMP1 were attributed not only to the higher G6PDH and enzymes involved in the oxidative defense such as superoxide dismutase (SOD) and catalase (CAT) but also to other key metabolic enzymes involved in lipid biosynthesis. This study provides an effective approach in developing the *Aurantiochytrium* sp. mutant with superior DHA production capacity that has the potential for industrial applications.

## Introduction

The global demand for docosahexaenoic acid (DHA, 22:6, n-3), an essential omega-3 polyunsaturated fatty acid, has increased tremendously over the past few years and is expected to keep on growing in the future (1). DHA is commonly marketed as dietary supplements, pharmaceuticals, infant formulas, and functional foods and has been extensively applied in aqua and animal feed to generate healthier, increased growth, and survival rate for farmed fish and animals (1). DHA has been reported to be vital for human health and well-being especially in supporting the development of the brain and eye of infants as well as curing or preventing several chronic diseases including hypertension, Alzheimer, arthritis, and adult-onset diabetes mellitus (2). In addition, a recent report showed that omega-3, particularly DHA, is able to decrease severity among patients with COVID-19 by lowering the production of proinflammatory cytokines that resulted in decreased viral entry and promoted better immune function (3). Yet, despite the importance, a recent study showed that more than 90% of the global population consumed less than the recommended optimal dose of DHA (0.25 g/day) in the diet (4). Oil extracted from marine fatty fish (often referred to as fish oil), such as Salmon and Tuna, is at present the major source of DHA (4). The expanding demands for DHA worldwide are now placing pressure on both fisheries and the fish oil supply, and it is expected to be unable to meet the market demand soon. This is due to declining of the fatty fish sources due to overfishing as well as other environmental factors. There are also possible health risks associated with the consumption of fish oils, such as food poisoning and allergies, particularly when there are issues of seawater contamination or toxicity and outbreaks of fish diseases (4). Considering the disadvantages of fish-oil-based DHA and for better commercial gains, efforts have been made to find alternative sustainable sources for omega-3 fatty acid production.

Microbial oil, being produced in a controlled environment, is one of the current topics of massive research due to its sustainability and advantages compared with fish oil (5). Thraustochytrids such as *Aurantiochytrium, Schizochytrium*, and *Thraustochytrium* are marine heterotopic protists, commonly known as marine microalgae, which are excellent DHA producers as they are capable of producing up to 35–55% DHA from the total fatty acids (TFAs) (6). Furthermore, DHA from thraustochytrids has also been proven to be safe for human consumption as it is free from the common algal toxins such as domoic acid and prymnesin produced by some members of its kingdom Chromista (7). Therefore, its applications as a novel food supplement and infant formula have been recommended in many countries throughout the world. However, the low DHA production yield remains to be the key limiting factor for large-scale DHA production from thraustochytrids.

Although various strategies have been developed, stress-based strategies coupled with oxygen-rich culture conditions have been shown to be the most effective approaches in enhancing the lipids well as DHA biosynthesis in thraustochytrids (8–10). However, this strategy often resulted in the inevitable formation of a high level of reactive oxygen species (ROS) causing severe oxidative damage that resulted in the loss of protein function as well as peroxidation of lipid (11, 12). Lipids, particularly PUFA, are highly susceptible to oxygen radical attack, and the resulting oxidative species are detrimental to cell metabolism and limit lipid productivity (13). Several studies have found that mitigation of oxidative stress through the addition of antioxidants, strain improvement through adaptive laboratory evolution (ALE), and overexpression of the genes involved in oxidative defense has significantly improved the DHA and PUFA content in thraustochytrids (12, 14–16). Furthermore, enforcing the pool of precursors for lipid biosynthesis, particularly the Acetyl-CoA and NADPH, has also been another promising strategy to enhance lipid and DHA production in thraustochytrids (17–20). In thraustochytrids, the supply of Acetyl-CoA pool is achieved by the action of ATP-citrate lyase (ACL) that cleaves citrate to generate acetyl CoA and oxaloacetate. The subsequent conversion of acetyl CoA into fatty acids requires a high level of NADPH as the principal reductant which is predominantly supplied by malic enzymes (ME) and glucose 6-phosphate dehydrogenase (G6PDH) (21). However, studies by Cui et al. (19, 22) found that the NADPH supply for the FAS pathway (which is responsible for saturated fatty acid production in thraustochytrids) and polyketide pathways (responsible for PUFA production) might be specific where overexpression of ME, as well as the G6PDH, has been found to enhance the production of saturated fatty acids and PUFA content in *Aurantiochytrium* sp. YLH70, respectively. Thus, the generation of mutants that possess both high oxidative tolerance and G6PDH activity is hypothesized to be able to enhance the DHA production in thraustochytrids.

In this study, we aimed to generate *Aurantiochytrium* SW1 mutant with high tolerance toward oxidative stress and high G6PDH activities through plasma mutagenesis coupled with chemical screening strategies. Plasma mutagenesis is one of the most efficient techniques in generating extensive mutation in microbes as it has been proven to poses high bioavailability, high energy density, poor repair effects, and good spatial resolution of energy deposition compared with traditional radiation sources (e.g., UV, γ-, and χ-rays) (23). It has already been successfully employed for mutation breeding of different microorganisms including bacteria (24), fungi (25), and microalgae (26, 27). For increased efficiency in generating mutant with the desired traits, the mutant generated after the plasma mutagenesis will be then challenged with zeocin and polydatin, generating mutant with superior oxidative stress G6PDH activity. Zeocin is a bleomycin-family glycopeptide antibiotic (28), a potent antibiotic that significantly inhibits the growth of thraustochytrids by generating a pseudo enzyme that interacts with oxygen and creates radicals free of superoxide and hydroxide which instigate lipid peroxidation and other cellular molecule oxidations, resulting in cellular damage. In contrast, polydatin (3,4,5-trihydroxystilbene-3-β-d-glucoside; transresveratrol 3-β-mono-D-glucoside; and piceid) is a chemical compound present in polygonum cuspidate and other plants that have been reported to inhibit G6PDH, the key enzyme for pentose phosphate pathway (29). This study provides the first report that integrates plasma mutagenesis coupled with zeocin and polydatin-based screening strategy, to generate mutants with superior tolerance to oxidative stress as well as high G6PDH activity for enhanced DHA production by thraustochytrids.

## Materials and Methods

### Organism and Culture Conditions

The starting microorganism used in this study is *Aurantiochytrium* sp. SW1 (GenBank: KF500513), provided by Microbial Physiology Lab, School of Biosciences and Biotechnology, Universiti Kebangsaan Malaysia as well as Colin Ratledge Centre for Microbial Lipid, Shandong University of Technology and has been deposited in UNiCC UPM under the accession number of [UPMC 963]. This organism was maintained on seawater nutrient agar (SNA) as slant culture which contained 28 g/L nutrient agar and 17.5 g/L artificial seawater accounting for 50% (w/w) salinity. Seed cultures were prepared by inoculating 100 ml of a seeding broth with a strip of SNA slant agar containing ~10 colonies of 48 h-old SW1 cells in 500 ml Erlenmeyer flasks. Seed cultures were then incubated at 28°C for 48 h with an agitation rate of 200 rpm. The medium used in seed cultures contained 60 g/L glucose, 2 g/L yeast extract, 10 g/L monosodium glutamate (MSG), and 6 g/L artificial sea salt. The composition of sea salt used in this study was described by Manikan et al. (6). Seed culture with an inoculum size of 10% (v/v) was then inoculated into the production medium for further study. There were two different media used as the production medium; (1) Burja which contained similar composition of the seed media and (2) glucose, peptone, and tryptone (GPT) that is also contained similar composition of glucose and sea salt as the seed media but the nitrogen sources (MSG and yeast extract) were replaced to peptone and tryptone (8+4 g/L, respectively).

### Determination of SW1 Sensitivity Toward Zeocin and Polydatin

Fifty microliters of SW1 culture in the exponential phase (36 h) was spread on SNA plates containing 0, 5, 10, 15, 20, 25, or 30 μM/ml of zeocin as well as 0, 50, 100, 200, 300, 400, 500, and 600 μM/ml of polydatin and incubated at 28°C for up to 7 days. However, for SNA plates with zeocin, only 2 g/L pf artificial sea salt was added as zeocin was sensitive toward high salt concentration. A graph of the lethal rate vs. concentration of the chemicals was plotted. A minimal three replicate plates were used for each condition.

### Plasma Mutagenesis and Screening of the Potential Mutant

Before mutagenesis, the SW1 cells were cultivated in a 500 ml shake flask containing 100 ml production medium at 28°C, 200 rpm for 36 h and then diluted to OD600 value between 0.6 and 0.8 with 0.2 mol/L sterile phosphate-buffered saline (PBS) buffer (pH 7.4). To protect cells from lysis caused by water evaporation, glycerol (10% v/v) was added to the cell suspension. A 20-μl aliquot of the above suspension was applied to a sterilized sample plate. The sample plate was later exposed to plasma radiation for a given time under the operating parameters of; radio frequency power input of 100 W, helium flow rate of 10 L/min, and plasma action distance of 2 mm. After mutagenesis, the cells were directly eluted with 1 ml sterile PBS buffer. After the appropriate dilution, 100 μl of the cell suspension was spread onto agar plates and incubated at 28°C for up to 7 days. The lethal rate was determined and optimized for optimal exposure time with the plasma radiation. Then, using the optimal lethal rate, the radiated SW1 cells were then spread on the agar containing zeocin (incubated in dark) and incubated at 28°C for up to 7 days. The colony that survived was then streaked on the agar containing polydatin, and the colonies that grew on the plate were selected for further analysis.

### Comparative Analysis of the Wild Type (SW1) and the Mutant Strains (YHPM1)

Comparative analysis on the growth, lipid, fatty acids, antioxidant enzymes, stress marker, and other key metabolic enzymes of the wild type (SW1) and the mutant strains (YHPM1) was carried out in a 500-ml shake flask, containing 100 ml cultivation media for 48 and 96 h of cultivation. The two different production media used during the screening process are as mentioned in the “Organism and Culture Conditions” section. The dry cell weight, lipid, and fatty acid compositions were determined according to the “Determination of dry cell weight, lipid extraction, and fatty acid analysis” section.

### Cell-Free Extract Preparations and Enzyme Assays

The mutant and the WT cells were harvested using centrifugation and were then suspended in an extraction buffer. Then, the cells were subsequently disrupted by ultrasonication (Scientz-II D sonifier, Ningbo Scientz Biotechnology Co., Ltd. CHN) at 400 W × 5 s with cooling in between on ice for 10 min (30). The cells were then centrifuged at 12,000 × *g* for 10 min at 4°C using Eppendorf centrifuge 5810R, and the supernatant was filtered through the Whatman No. 1 filter paper to recover the cell-free extract. The supernatant containing cytoplasmic and mitochondrial enzymes was subjected to enzyme activity analysis. The activities of four enzymes, namely, malic enzyme (ME), ATP: citrate lyase (ACL), glucose-6-phosphate dehydrogenase (G-6-PDH), and NADP^+^-isocitrate dehydrogenase (ICDH) were determined using continuous assays following the oxidation and reduction of NAD(P)(H) at 340 nm and 30°C. The change in absorbance was followed continuously for 10 min using software (UVPROBE2.31). Specific activity is expressed as nmol/min·mg protein. Superoxide dismutase (SOD) and catalase (CAT) activities were determined using an assay kit (Beyotime Institute of Biotechnology, Shanghai, China) according to the manufacturer's instructions. The specific activity (U/mg protein) was defined as the activity unit/mg protein. Protein concentration was determined using the Bradford method with bovine serum albumin (BSA) as a standard (31).

### ROS Determination

The intracellular ROS levels were determined using the Reactive Oxygen Species Assay Kit (Beyotime Institute of Biotechnology, Shanghai, China) according to the manufacturer's instructions. In brief, 1 ml of SW1 cultures were harvested and washed with PBS buffer. A diluted dichlorodihydrofluorescein diacetate (DCFH-DA) probe was then added into the cell suspension and incubated at 37°C for 30 min. The excess probe was washed twice with PBS buffer to ensure only the intracellular fluorescence was measured. Fluorescence intensity was detected using a fluorescence spectrophotometer with the excitation and emission wavelengths at 485 and 535 nm, respectively.

### Lipid Peroxidation Assay (Malondialdehyde Assay)

The lipid peroxidation level was determined by measuring the malondialdehyde (MDA) equivalent according to the method proposed by Heath and Packer (32). In brief, the harvested cells of 1 ml were disrupted, homogenized in 5% (w/v) trichloroacetic acid (TCA), and then centrifuged at 10,000 × *g* for 10 min at 4°C. The supernatant was then mixed with 0.67% (w/v) thiobarbituric acid (TBA) solution and boiled for 20 min. The absorbance of the reaction mixture was recorded at 532 and 600 nm. The absorbance value recorded at 532 nm was subtracted by the non-specific absorbance value recorded at 600 nm, and the MDA equivalent content was measured using the 155 mM/cm extinction coefficient.

### Cultivation of YHPM1 and SW1 in 5L Bioreactor

The YHPM1 and SW1 growth, lipid, and DHA production capacity were compared using carried 5-L bench-top bioreactors (Minifors-Infors HT) with 3 L working volume with the utilizing GPT media as described in the “Comparative analysis of the wild type (SW1) and the mutant strains (YHPM1)” section. The temperature of the culture was controlled at 28°C, and the impeller speed was fixed at 500 rpm with the aeration of 1 vvm. Samples were then collected every 12 h for 120 h to determine the biomass, lipid, DHA, and residual sugar concentration.

### Analytical Methods

#### Analysis of Glucose

The supernatant obtained from the centrifugation of the biomass was utilized to measure the residual glucose content, using the glucose oxidase kit according to a previous study (33, 34).

### Determination of Dry Cell Weight, Lipid Extraction, and Fatty Acid Analysis

The dry cell weight of SW1 was determined according to the previous method (30). Lipid extraction was performed using chloroform–methanol (2:1, v/v), as described by Folch et al. (35). The extract was vaporized at room temperature and dried in a vacuum desiccator until a constant weight was attained. Fatty acid compositions of the samples were determined as fatty acid methyl esters (FAMEs) by gas chromatography (HP 5890) equipped with a capillary column (BPX 70, 30 cm, 0.32 μm). FAME was prepared by dissolving 0.05 g of the sample in 0.95 ml hexane, and the mixture was added to 0.05 ml of 1 M sodium methoxide. The injector was maintained at 200°C. Then, 1 μl of the sample was injected using helium as a carrier gas with a flow rate of 40 cm^3^min^−1^. The temperature of the GC column was gradually increased at 7°C min^−1^ from 50 (5 min hold) to 200°C (10 min hold). Fatty acid peaks were identified using Chrome Leon Chromatography software (Dionex, Sunnyvale, California, USA). FAMEs were identified and quantified by comparison with the retention time and peak areas of SUPELCO FAME standard (Bellefonte, PA, USA).

### Statistical Analysis

SPSS 16.0 (SPSS Inc. Chicago, IL, USA) software was used to perform the statistical analysis with three independent replicates (*n* = 3), and the data obtained from the experiments were used to calculate the mean values and the standard errors. The differences between means of the test were calculated using the Student's *t*-test, and *p* < 0.05 was considered significantly different.

## Results

### Mutagenesis With Plasma Radiation

To determine the optimal exposure time of the plasma radiation, the SW1 cells were treated with plasma radiation for varying durations (i.e., 0, 10, 20, 30, 40, 60, 80, or 100 s), and the cell was then incubated at 28°C for up to 7 days. The result showed that the optimal treatment time was at the 80 s with the lethal rate of 98.0–99.0%. A high lethal rate was used in this study to generate mutants with extensive mutation as well as avoid the lengthy screening process. According to previous studies, an extensive mutation level was typically achieved when the lethal rate was above 90% (36). Besides, a superior strain of *Bacillus subtilis* was generated after plasma mutagenesis treatment with a 95% lethal rate (24).

### Screening of Mutant High Oxidative Tolerance and G6PDH Activity

**Figure 1:**
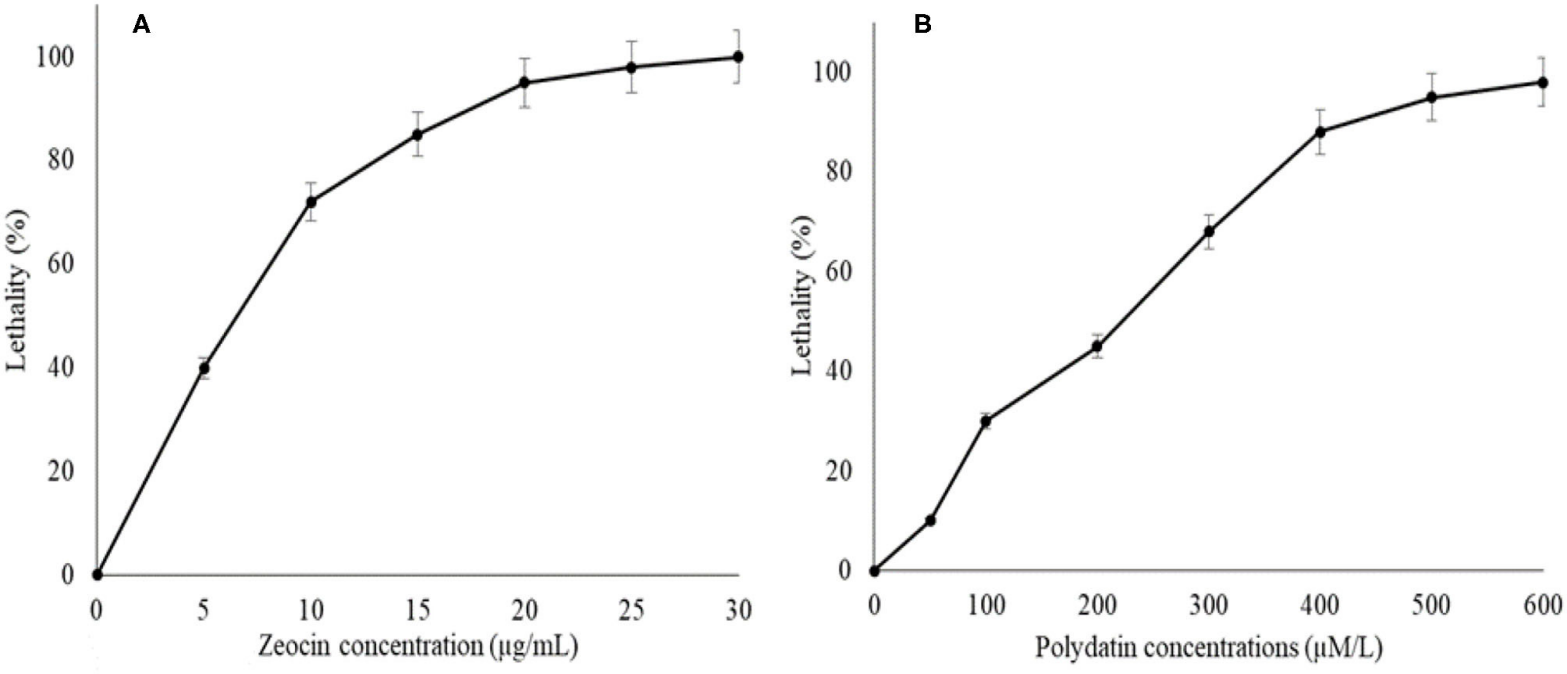
The lethal rates curves of SW1 cultured in agar plates containing; **(A)** 0, 5, 10, 15, 20, 25, and 30 μg/mL zeocin as well as **(B)** 0, 50, 100, 200, 300, 400, 500, and 600 μM/L of polydatin, respectively. Values and error bars represent the means and the standard deviations of triplicate experiments (*n* = 3).

The agar plates containing 0, 5, 10, 15, 20, 25, or 30 μM/ml of zeocin as well as 0, 50, 100, 200, 300, 400, 500, and 600 μM/ml polydatin were prepared to determine the inhibition levels of both chemicals toward SW1 (Figures 1A,B). The result showed that the inhibition levels of SW1 increased with the increase of zeocin and polydatin concentration, reaching ~95–98% with 20 μM/ml zeocin and 500 μM/ml polydatin, respectively. Thus, agar plates containing 20 μM/ml zeocin and 500 μM/ml polydatin were used to screen and isolate SW1 mutants with superior oxidative defense as well and G6PDH activity. After exposing SW1 to the plasma radiation, the SW1 cells were immediately spread on the agar plates containing 20 μM/ml of zeocin and incubated for 4–7 days. Only approximately 5 ± 1.25 colonies were observed on the zeocin plates, and then, all the five colonies were transferred to the polydatin plates and were further incubated for another 4–7 days. Interestingly, only one colony (named YHPM1) was able to survive on the agar plate containing 500 μM/ml polydatin. Thus, to evaluate and compare the cellular physiology of the mutant with the WT strain, a comparative study was conducted.

### Comparative Analysis on Cellular Morphology, Cell Growth, and Fatty Acid Biosynthesis as Well as Oxidative and Key Metabolic Enzymes of YHPM1 and SW1

**Figure 2:**
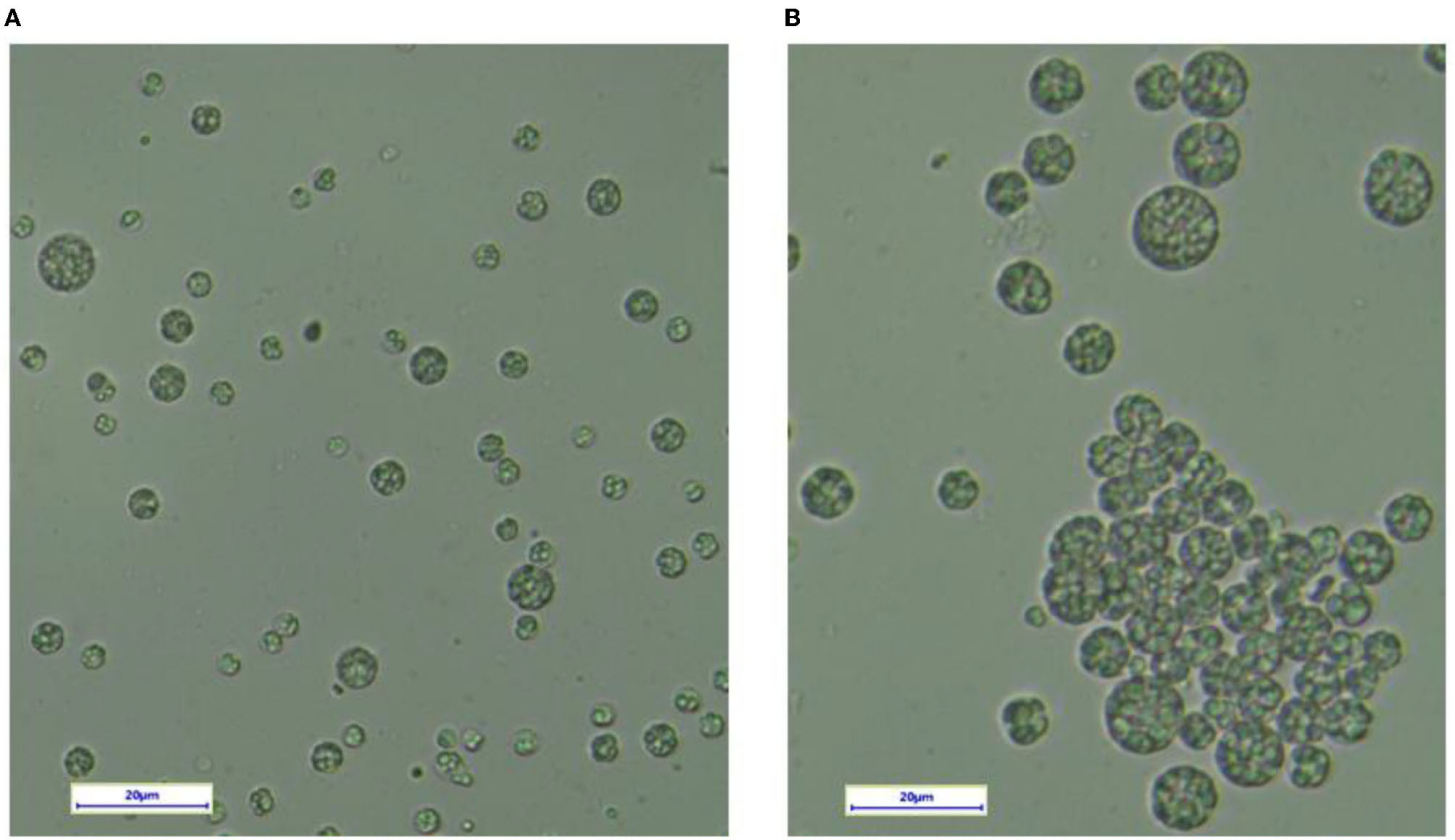
The cellular morphology of **(A)** SW1 and **(B)** YHPM1 under the light microscope. The scale bar represents 20 μm.

To evaluate the changes in the cellular morphology of YHPM1 over the wild type (SW1), both the cultures were cultivated in a 500-ml shake flask for 96 h of cultivation, and the differences in the cellular morphology were compared (Figure 2). The result showed that both strains exhibited significant differences in the cellular size where most of the YHM1 poses a relatively bigger in diameter (approximately between 5 and 20 μM) colonies in comparison to the WT (2–10 μM). Besides, the YHM1 cell was shown to be more intact with a compact and large lipid droplet within the cell in comparison to SW1, confirming the mutation effect at the morphological level (Figure 2).

**Figure 3:**
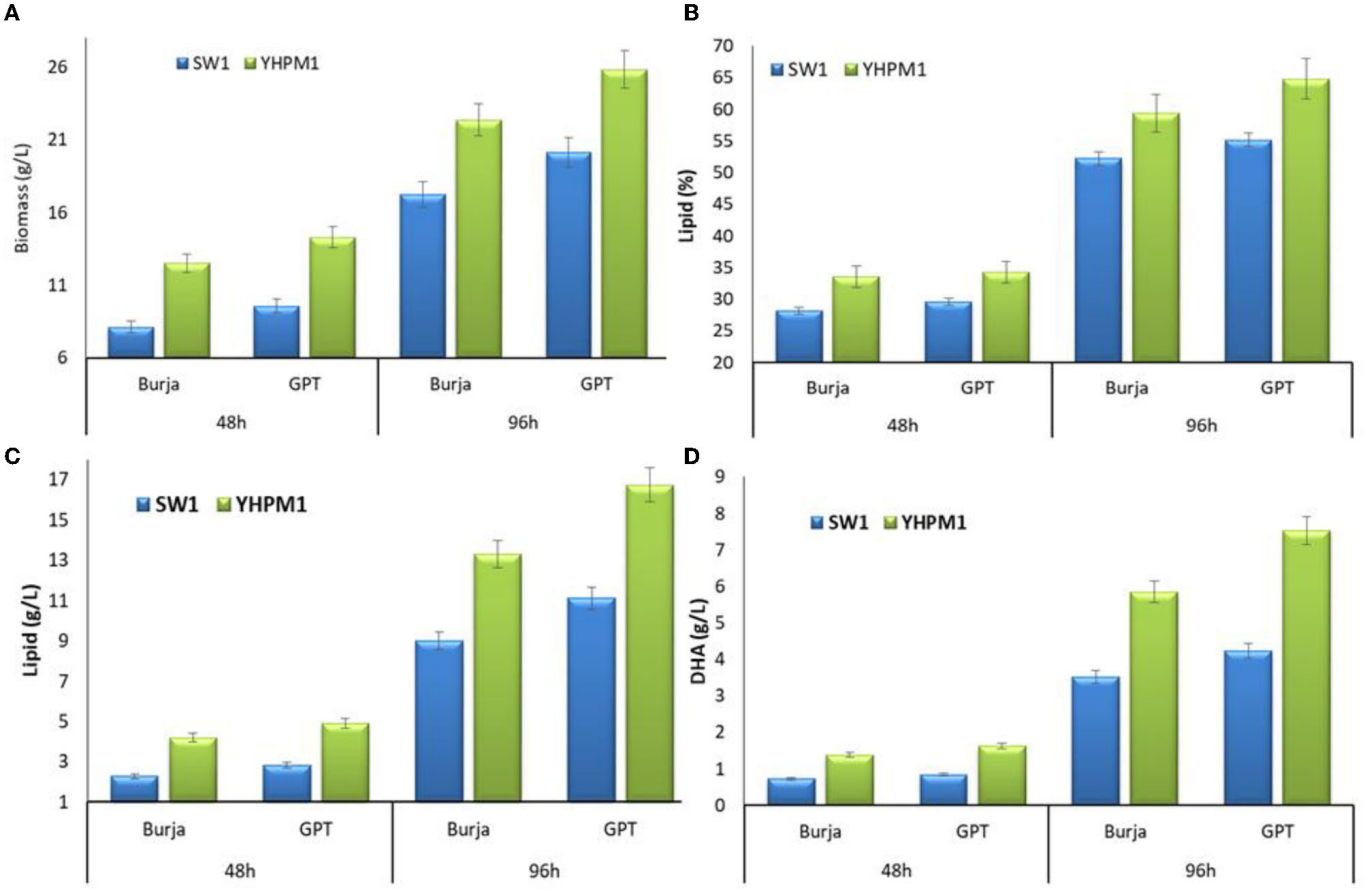
The **(A)** biomass, **(B)** lipid content (%), **(C)** lipid production (g/L) as well as **(D)** DHA production (g/L) of SW1 and YHPM1 cultured in 500 mL shake flask for 48 and 96 h of cultivation in Burja and GPT media. Values and error bars represent the means and the standard deviations of triplicate experiments (*n* = 3).

In addition, to further evaluate the mutation effect of YHPM1 over SW1, a comparative study on cellular growth, total lipid, and fatty acid biosynthesis capabilities was performed with two different media, namely Burja and GPT at 48 and 96 h of cultivations, respectively. As shown in Figure 3, YHPM1 exhibited superior growth in comparison with SW1 in both media and cultivation hours. The most pronounced impact was observed in GPT media where YHPM1 poses 18 and 43% greater biomass production at 48 and 96 h of cultivation, in comparison with SW1, respectively. Furthermore, the lipid content of YHPM1 reached 64.18% in GPT media at 96 h of cultivation where it is 22.27% higher than that of the WT. Furthermore, the total lipid production (g/L) of YHPMI was 67.42 and 47.19% higher than that of SW1 in GPT and Burja media, respectively.

Furthermore, a pronounced change in the fatty acid compositions was also observed in both strains. The YHPM1 strain was shown to poses a 15–27% higher PKS product which is mainly composed of the DPA and DHA in both media and cultivation hours in comparison to SW1 (Table 1). In addition, a much significant difference was observed in the total DHA production (g/L) where YHPM1 produced ~8.0 g/L DHA when grown with GPT media at 96 h of cultivation, 60% higher than that of SW1 (Figure 3D). These results indicated that the mutant strain exhibited not only a superior growth and lipid content but also PUFAs and DHA production.

**Table 1:** The fatty acid compositions of SW1 and YHPM1 cultured in 500 ml shake flask for 48 and 96 h of cultivation in Burja and GPT media.

**Fatty acids**	**Burja**				**GPT**			
	**48 h**		**96 h**		**48 h**		**96 h**	
	**SW1**	**YHPM1**	**SW1**	**YHPM1**	**SW1**	**YHPM1**	**SW1**	**YHPM1**
14:0	3.62 ± 0.17	3.94 ± 0.20	3.01 ± 0.11	3.36 ± 0.19	4.71 ± 0.26	3.48 ± 0.22	3.10 ± 0.17	2.25 ± 0.17
14:1	0.24 ± 0.01	0.21 ± 0.01	0.30 ± 0.01	0.19 ± 0.03	0.56 ± 0.03	0.49 ± 0.05	0.41 ± 0.03	0.33 ± 0.02
15:0	3.23 ± 0.16	2.24 ± 0.11	2.41 ± 0.09	1.91 ± 0.27	4.88 ± 0.27	4.64 ± 0.26	1.20 ± 0.07	1.23 ± 0.07
16:0	45.12 ± 2.17	43.56 ± 2.23	38.13 ± 1.45	33.83 ± 2.40	43.59 ± 2.40	42.97 ± 2.36	41.70 ± 2.91	36.12 ± 1.99
17:0	1.33 ± 0.06	0.95 ± 0.05	1.53 ± 0.06	0.74 ± 0.04	0.76 ± 0.03	0.51 ± 0.03	1.30 ± 0.07	1.02 ± 0.06
18:0	1.40 ± 0.07	1.34 ± 0.07	1.02 ± 0.04	1.08 ± 0.07	1.23 ± 0.09	1.06 ± 0.06	1.42 ± 0.08	1.34 ± 0.07
20:5n3 (EPA)	0.25 ± 0.01	0.31 ± 0.02	0.83 ± 0.03	0.75 ± 0.02	0.45 ± 0.03	0.41 ± 0.02	0.75 ± 0.04	0.79 ± 0.04
22:5n3 (DPA)	6.14 ± 0.29	7.10 ± 0.36	9.31 ± 0.35	11.35 ± 0.38	6.86 ± 0.40	7.78 ± 0.43	8.40 ± 0.46	10.24 ± 0.56
22:6n3 (DHA)	34.56 ± 1.66	38.79 ± 1.99	41.89 ± 1.59	45.92 ± 1.91	34.77 ± 1.74	36.60 ± 2.01	40.56 ± 2.88	44.81 ± 2.46
Other minor fatty acids	2.03 ± 0.10	1.52 ± 0.08	1.65 ± 0.06	0.87 ± 0.12	2.19 ± 0.10	1.71 ± 0.09	1.35 ± 0.13	1.85 ± 0.10
FAS products	59.05 ± 2.83	53.80 ± 2.76	47.97 ± 1.82	41.90 ± 3.19	57.92 ± 1.59	55.21 ± 3.04	50.29 ± 2.77	44.16 ± 2.43
PKS products	40.95 ± 1.97	46.20 ± 2.37	52.03 ± 1.98	58.10 ± 2.21	42.08 ± 2.33	44.79 ± 2.46	49.71 ± 2.15	55.84 ± 3.07

*Values represent the means of triplicate experiments (n = 3)*.

### Comparative Analysis on Oxidative Defense and Key Metabolic Enzymes of YHPM1 and SW1

**Figure 4:**
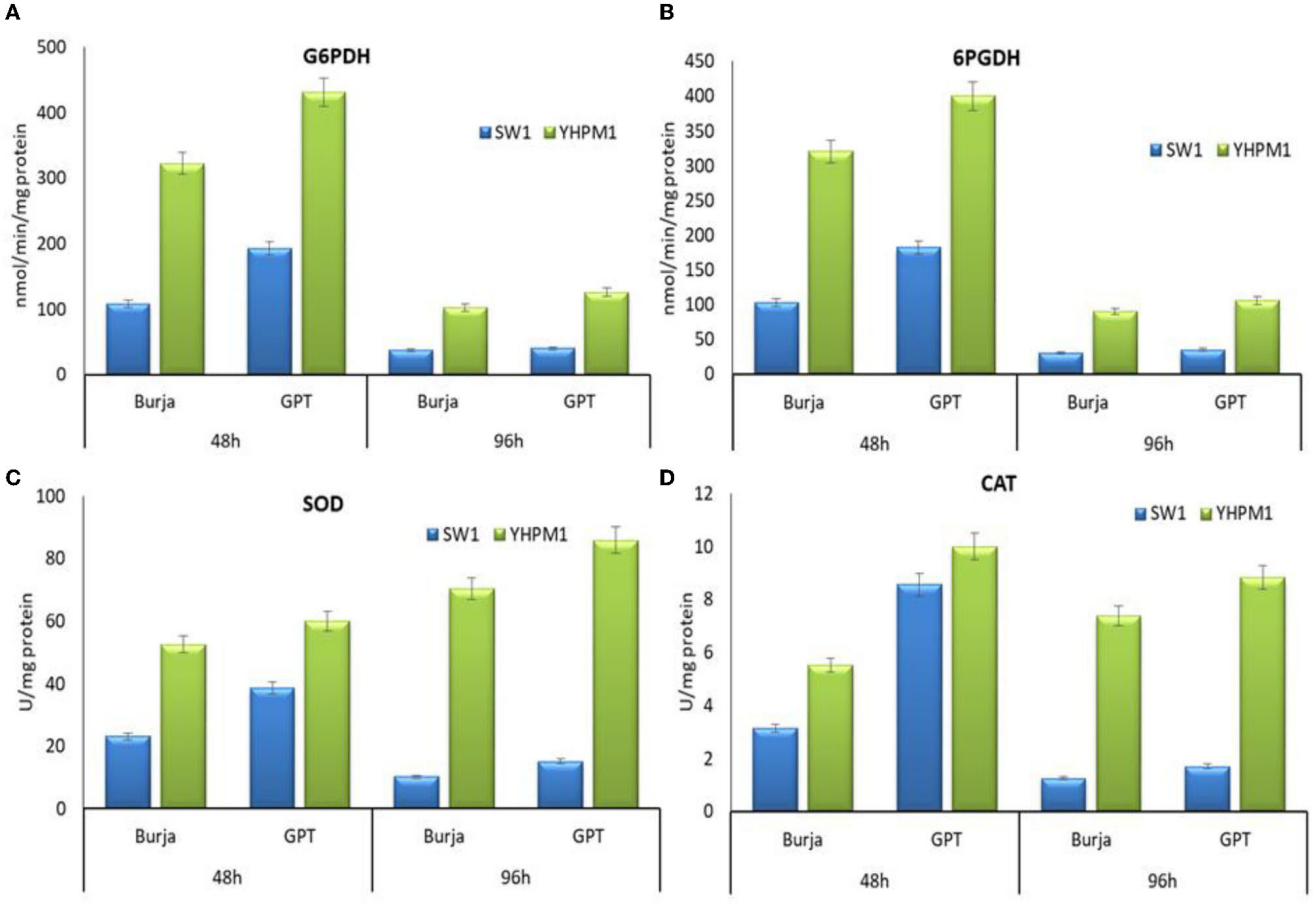
The activity of **(A)** G6PDH, **(B)** 6PGDH, **(C)** SOD as well as **(D)** CAT of SW1 and YHPM1 cultured in 500 mL shake flask for 48 and 96 h of cultivation in Burja and GPT media. Values and error bars represent the means and the standard deviations of triplicate experiments (*n* = 3).

To confirm the effects of polydatin- and zeocin-based screening, the activity of G6PDH, 6PGDH, as well as the antioxidant enzymes of the mutant (YHPM1) and wild type (SW1), was compared. The results showed that G6PDH and 6PGDH activities in YHPM1 were, in fact, higher than SW1 with the most significant increase found at 48 h of cultivation, achieving a nearly 2.2- and 1.8-fold higher than that of SW1, respectively (Figure 4). In addition, both the SOD and CAT activities inYHPM1 was also exhibited a 3–4-fold higher than that of SW1 especially in GPT media after 96 h of cultivation, consistent with its ability to grow at a high concentration of zeocin during the screening process.

**Figure 5:**
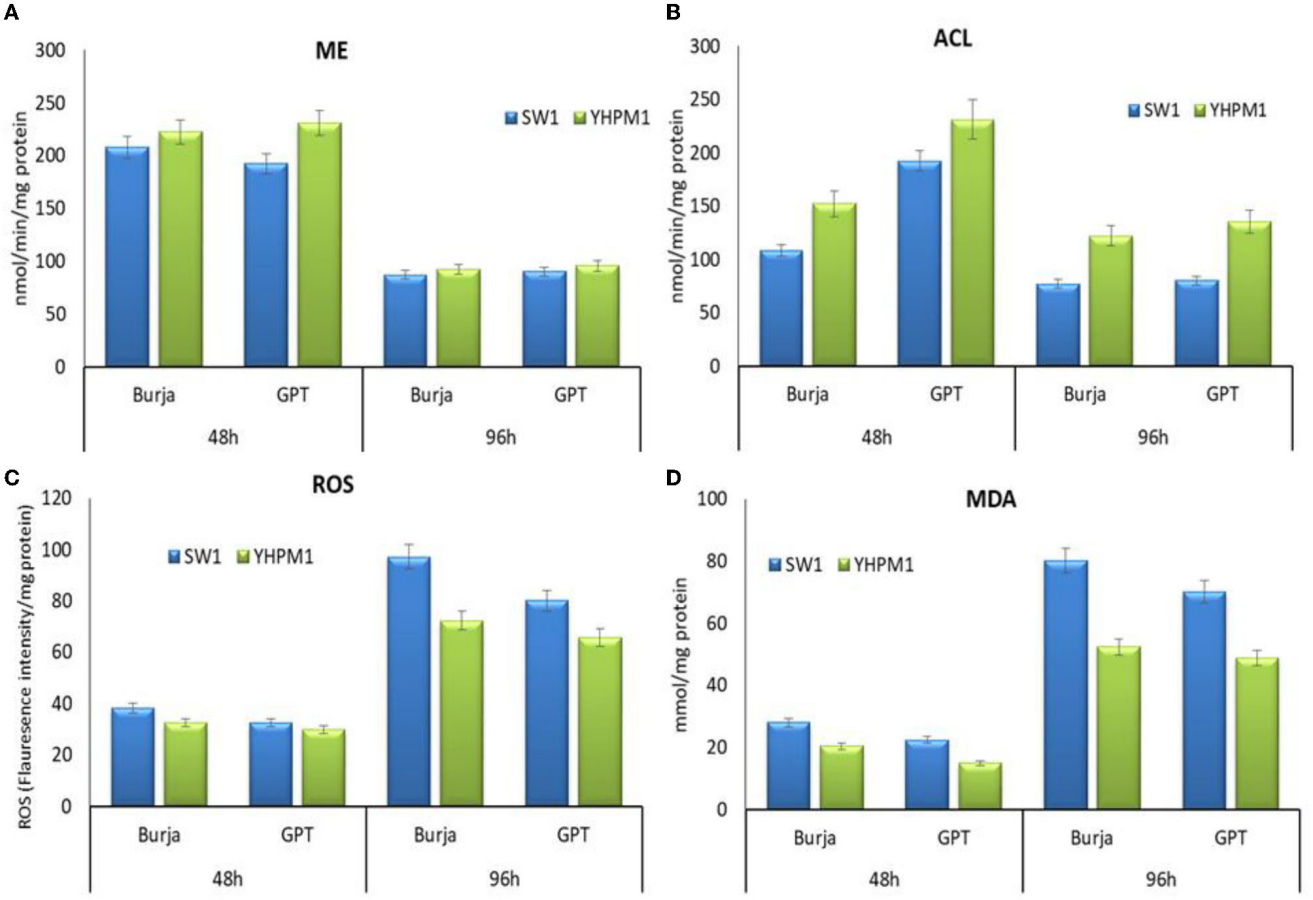
The activity of **(A)** ME, **(B)** ACL, **(C)** ROS as well as **(D)** MDA of SW1 and YHPM1 cultured in 250 mL shake flask for 48 and 96 h of cultivation in Burja and GPT media. Values and error bars represent the means and the standard deviations of triplicate experiments (*n* = 3).

In addition, the impact on the other key enzymes involved in lipogenesis was also elucidated. A pronounce impact was also observed in ACL activities where YHPM1 exhibited a 68.12% higher activity compared with SW1 at 96 h of cultivation in the GPT media. Nevertheless, the activity of ME in YHPM1, although higher at 48 h, but not significant at 96 h of cultivation in comparison with SW1, indicating the significant elevation in the total lipid content, was attributed to the higher NADPH supply from G6PDH and 6PGDH, which explains the significant improvement in the PUFA content of YHPM1. Furthermore, the oxidative stress indicators of YHPM1, ROS, and MDA were also shown to be 25–53% lower than that of SW1 especially at the 96 h of cultivation, mainly attributed to the stronger antioxidant aptitudes poses by YHPM1 in comparison with SW1 (Figure 5).

Based on this result, it is indicated that YHPM1 has higher overall growth, lipid, and DHA production capacity as compared with SW1 in the shake flask level. To evaluate its potential for industrial-scale application, an upscaling experiment at a 5 L bioreactor scale was conducted.

### Cultivation of YHPM1 and SW1 in 5 L Bioreactor

**Figure 6:**
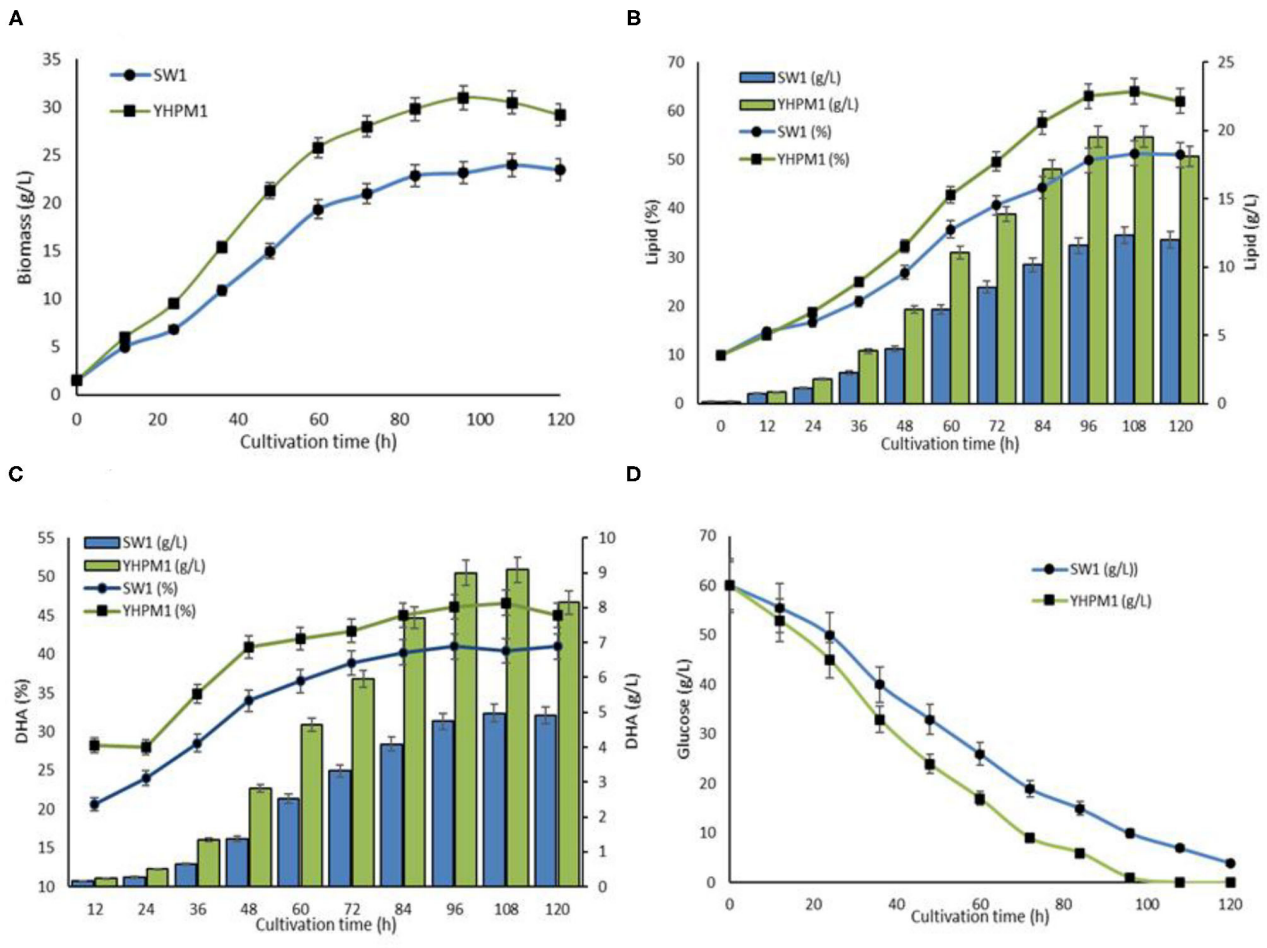
**(A)** Biomass, **(B)** lipid, **(C)** DHA, and **(D)** glucose consumption rate of YHPM1 and SW1 cultivated in 5L bioreactor for 120 h.

A comparative study of SW1 vs. YHPM1 was conducted to evaluate the growth, lipid, and DHA production capacity in a larger fermentation scale, by utilizing GPT media as described in the “Cultivation of YHPM1 and SW1 in 5L bioreactor” section (Figure 6). The result showed that YHPM1 exhibited a 30, 65, and 80% higher overall biomass, lipid, and DHA production in comparison with SW1, respectively (Figures 6A–C). Besides, YHPM1 also exhibits a faster substrate utilization rate (Figure 6D) as compared with that of the WT, a characteristic that is comparable with the other high DHA producing strains of thraustochytrids such as *Schizochytrium* sp. HX308 (37), *Schizochytrium* sp.31 (38), and *Aurantiochytrium* sp. SR21 (39). Thus, a mutant strain of *Aurantiochytrium* sp. that has a promising potential for industrial-scale DHA production has been successfully developed in this study.

## Discussion

Thraustochytrids are unique due to its ability to produce high lipid and DHA content from its DCW, but the low volumetric DHA production limits its application at the industrial scale. Thus, many studies have been conducted to enhance the DHA production yields from thraustochytrids (8–10). One of the factors attributed to the high oleaginousity of thraustochytrids is due to the fact that there are two distinct pathways involved in the biosynthesis of fatty acids in this organism: the type I fatty acid synthase (FAS) pathway and a polyketide synthase (PKS) pathways (40). It was later found that only the PKS pathway is responsible for the DHA biosynthesis in thraustochytrids (41). Thus, efforts have been made to enhance DHA production by overexpressing the genes involved in PKS pathways as well as elucidating the specific NADPH supplier for the PKS pathways (17, 19). Studies by Cui et al. (19) showed that the overexpression of G6PDH activity significantly enhanced PUFA production in thraustochytrids, thus, indicating that the NADPH for the PKS pathway is specifically channeled by the G6PDH. Aside from the NADPH, it is also important to develop strategies to mitigate the oxidative stress in the thraustochytrids, since lipids, particularly PUFA, are highly susceptible to oxygen radical attacks that limit the DHA productivity (13). Thus, the generation of mutants with high oxidative tolerance and G6PDH activity would be one of the most efficient ways to increase the DHA production by thraustochytrids, including the *Aurantiochytrium* sp.

At present, the advancement in microbial genetic engineering and synthetic biology has led to a significant increase in super strain development for the production of important metabolites of interest, including PUFAs from the microalgae (17, 42, 43). Nevertheless, genetic engineering technology in *thraustochytrids* including the *Aurantiochytrium* sp. is still in its infancy as the development of a transformation system was only realized by Sakaguchi et al. (44). Furthermore, due to the uncertain and complex metabolic regulation network of *Aurantiochytrium* sp., it was found that changes in one or two genes do not warrant producing transformants with the desired trait. For example, although the overexpression of G6PDH gave a promising impact on the PUFA and DHA content of *Aurantiochytrium* sp. SD116, the growth was significantly compromised, thus reducing the overall DHA production (g/L) compared with the WT strain (19). Therefore, the selection of suitable mutagenesis approaches remains the major challenge in producing the thraustochytrid mutant with desirable traits.

Plasma mutagenesis can directly mutate the *Aurantiochytrium* sp. strains by penetrating the cell wall with high transfer values of linear energy, thus, creating a stronger mutation effect (45). ROS produced by plasma can alter the structure and permeability of the cell membrane and interact with DNA and other macromolecules to change the cell metabolic activity and genetic characteristics, triggering the SOS repair mechanism, which can produce different kinds of mismatch sites (45). Furthermore, this technique has been proven to create more mutation sites than other techniques. Therefore, in this study, plasma radiation was deployed to generate mutants with superior growth and DHA biosynthetic capacity. Nevertheless, this technique is not without flaw as the process of obtaining mutants with the desired traits is difficult to realize due to random mutation impact. Thus, to obtain mutants with the desired traits, it is still crucial to develop proper and efficient screening methods after the mutagenesis process. Most conventional screening methods, such as phenotypic screening, are time and labor-consuming (36). Since certain chemicals are known to inhibit certain pathways which may result in impaired cellular metabolism of cells, it can be used to be the selective pressure to screen thraustochytrid mutant with desired traits (27).

In this study, zeocin and polydatin are the chemicals that were selected to be used as a selective pressure for the mutant generated through plasma mutagenesis. Zeocin is an antibiotic that is highly noxious to thraustochytrids where it instigates high ROS production in the cell, resulting in cell death (36). Zeocin with a concentration of 20 μg/ml that resulted in a 98% lethal rate on the WT strain was selected as the selective pressure for the mutant generated through plasma mutagenesis, obtaining mutant with high oxidative tolerance. In contrast, 500 μg/ml polydatin, which showed a 95% inhibition rate to the WT strain, was selected as the elective pressure to generate mutant with high G6PDH activity. This led to the identification of the YHPM1 strain, where this mutant is able to withstand the toxic effect of both chemicals used during the screening process.

The mutation effect was initially confirmed by cellular morphology, where YHPM1 poses significantly bigger cells, ranging from 5 to 20 μM, differing from the common size of *Aurantiochytrium* sp. which ranges from 2 to 10 μM in diameter (6). This observation was also in line with what was reported by Geng et al. (46) who found that the higher biomass and fatty acid content of the engineered strain *Schizochytrium* sp. HX-308 was followed by a much intact cellular morphology in comparison with the WT. Furthermore, analysis from the fatty acid content also reveals some insight into the mutation effect as YHPM1 and SW1 pose altered composition of major fatty acids, especially C14:0, C15:0, C16:0, C22:n5 (DPA), and C22:n6 (DHA). YHPM1 was found to poses higher content of DPA and DHA, as well as the overall PUFA content, as compared with SW1 probably due to more active PKS machineries than the FAS.

In addition, the mutation effect was further confirmed by determining the changes in the key metabolic enzymes compared with the WT. There was a clear change in the activity of the antioxidant enzymes in both of the strains. The SOD and CAT activities of the mutant (YHPM1) were ~3–4-fold higher than that of the WT, confirming the effect of zeocin screening. Zeocin works on cells by chelating metal ions (primarily iron) to generate radicals free of superoxide and hydroxide, which instigate lipid peroxidation and other cellular molecule oxidations, resulting in cellular damage (28). YHPM1 was capable to survive the lethal concentration of zeocin during the screening process as it poses a higher antioxidant capacity compared to the others. *Aurantiochytrium* sp. strain with a good antioxidant capacity is one of the important criteria as it correlates to its higher capacities to produce DHA and circumvent lipid peroxidation, which is one of the major causes of PUFA and DHA degradations (16). Several studies have shown that enhancement of the oxidative defense has a positive correlation with the increase of PUFA content in microalgae, particularly the thraustochytrids (11, 12). For example, the addition of antioxidants such as ascorbic acid (14) and sesamol, as well as overexpression of SOD in *Schizochytrium* sp. (16), have successfully alleviated the oxidative stress and increased the DHA content in the microalgae by elevating the total antioxidant capacity as well as lowering the ROS and lipid peroxidation in the cells. Lipids, particularly PUFA, are highly susceptible to oxygen radical attack, and the resulting oxidative species are detrimental to cell metabolism and limit lipid productivity (13).

Aside from posing a higher antioxidant capacity, YHPM1 was also shown to have significantly higher activity of the G6PDH and 6PGDH activities (Figure 3). Higher G6PDH activity has been implicated with more active PKS machineries as it has been proposed to be responsible for the NADPH supply in the PKS pathway of *Aurantiochytrium* sp. (19). Nevertheless, no inhibition of growth was observed in this mutant strain, differing from the previous study which reported that the overexpression of G6PDH resulted in compromised growth, despite the positive effect on the PUFA and DHA contents (19). Furthermore, the activity of other key enzymes especially the ACL was also significantly higher in YHPM1 in comparison to the wild type (SW1). From the activity of the key enzymes, it was demonstrated that YMPM1 generates a higher provision of NADPH as well as acetyl-CoA compared with SW1, explaining its higher lipid and DHA biosynthesis capacity.

In the effort to enhance the DHA production by thraustochytrids, numerous metabolic engineering strategies have been developed (Table 2). It was found that the lipid and DHA contents, as well as the DHA biosynthetic capacity of YHPM1 generated in this study were comparable with the other high DHA yielding thraustochytrids, indicating that YHPM1 has a promising potential for lab and large-scale DHA production.

**Table 2:** Different metabolic engineering strategies to enhance docosahexaenoic acid (DHA) production from thraustochytrid strains.

**Thraustochytrids strains**	**Strategy**	**Lipid content (%, g/g biomass)**	**DHA content (% of total fatty acids)**	**DHA biosynthetic capacity (g DHA/g Biomass)**	**References**
*Schizochytrium sp. ATCC 20888*	Overexpression of ACL and ACC genes from *Schizochytrium* sp.	73.0%	38%	0.28	(47)
*Schizochytrium* sp. PKU#Mn4	Overexpression of the antioxidative gene superoxide dismutase from *Schizochytrium* sp.	35.6%	41%	0.15	(16)
*Aurantiochytrium* sp. SD116	Heavy-ion irradiation technique coupled with then two-step ALE: low temperature-based ALE and ACCase inhibitor quizalofop-p-ethyl based ALE	60%	52.6%	0.31	(48)
*Schizochytrium* sp. PKU#Mn4	ARTP mutagenesis coupled with the acetyl-CoA carboxylase (ACCase) inhibitor (clethodim)-based screening	45%	42%	0.18	(27)
*Aurantiochytrium* sp. YHPM1	Plasma mutagenesis coupled with zeocin and polydatin screening	61%	45%	0.30	This study.

## Conclusion

A mutant strain of *Aurantiochytrium* sp. (YHPM1) has been successfully developed through plasma mutagenesis coupled with zeocin and polydatin screening strategies, which possess a significantly higher DHA biosynthetic capacity in comparison with the parental strain. In addition, the potential mechanism for the superior growth, lipid, and DHA biosynthetic capacity of the mutant strain, compared with the parent strain, was also elucidated. This study provides the first report that integrates plasma mutagenesis coupled with zeocin- and polydatin-based screening strategies, to generate mutants with superior tolerance to oxidative stress as well as high G6PDH activity for enhanced DHA production by thraustochytrids.

## Data Availability Statement

The raw data supporting the conclusions of this article will be made available by the authors, without undue reservation.

## Author Contributions

YN conceived the study, designed and performed the experiments, interpreted the data, and drafted the manuscript. HH and PP participated in the experimentation. HM and TN data analysis. AA and YS participated in study design, supervised all experimental procedures, and finalized the manuscript. All authors contributed to the article and approved the submitted version.

## Conflict of Interest

The authors declare that the research was conducted in the absence of any commercial or financial relationships that could be construed as a potential conflict of interest.

## Publisher's Note

All claims expressed in this article are solely those of the authors and do not necessarily represent those of their affiliated organizations, or those of the publisher, the editors and the reviewers. Any product that may be evaluated in this article, or claim that may be made by its manufacturer, is not guaranteed or endorsed by the publisher.
